# The microbiome analysis of ripen grape berries supports the complex etiology of sour rot

**DOI:** 10.3389/fmicb.2024.1450443

**Published:** 2024-11-07

**Authors:** Chiara Brischetto, Vittorio Rossi, Giorgia Fedele

**Affiliations:** ^1^Department of Sustainable Crop Production (DI.PRO.VE.S.), Università Cattolica del Sacro Cuore, Piacenza, Italy; ^2^Research Center on Plant Health Modelling (PHeM), Università Cattolica del Sacro Cuore, Piacenza, Italy

**Keywords:** *Vitis vinifera*, minor grape rots, bunch microflora, high-throughput sequencing, acetic acid bacteria, Enterobacteriaceae, non-*Saccharomyces* yeasts

## Abstract

Sour rot (SR) is a grapevine disease complex that is not completely understood in its etiology and epidemiology. Recently, SR has received special attention due to its increasing economic importance due to crop losses and reduced wine quality. In this study, the fungal and bacterial microbiota of healthy (i.e., without rot symptoms) and rotten (i.e., exhibiting visual and olfactory SR symptoms) ripe bunches were characterized across 47 epidemics (39 vineyards in six Italian grape-growing areas) over three years. The 16S rRNA gene, ITS high-throughput amplicon sequencing, and quantitative PCR were used to assess the relative abundance and dynamic changes of microorganisms associated with SR. The estimators of genera richness of fungal communities within samples indicated a significantly different diversity between healthy and rotten bunches. For bacterial communities, the healthy and rotten bunches significantly differed in the total number of species, but not in abundance distribution across species. The bunch status (i.e., healthy and rotten) was a significant source of diversity (*p* < 0.01) when the community composition between samples was evaluated, indicating that microbiome composition varied between healthy and rotten bunches. In particular, healthy and rotten bunches shared 43.1 and 54.8% of fungal and bacterial genera, respectively; 31.3% (fungal) and 26.2% (bacterial) genera were associated with rotten bunches only. The yeast genera *Zygosaccharomyces*, *Zygoascus*, *Saccharomycopsis*, *Issatchenkia*, and *Pichia* and the bacterial genera *Orbus*, *Gluconobacter*, *Komagataeibacter*, *Gluconacetobacter*, and *Wolbachia* were strongly associated with bunches showing SR symptoms based on a linear discriminant analysis. These microorganisms have been associated with *Drosophila* insects in literature. The relationships between the microflora associated with SR-affected bunches and the roles of *Drosophila* in SR development need further investigation, which may open perspectives for more effective disease control.

## 1 Introduction

Grapes are affected by some diseases, collectively known as bunch rots, which affect bunches during ripening (Pearson and Goheen, 1988). *Botrytis* bunch rot (or gray mold) is caused by *Botryotinia fuckeliana* and is undoubtedly the most widespread rot. The non-*Botrytis* rots are sour rot (SR) (caused by a complex of microorganisms), ripe rot (caused by *Colletotrichum* spp.), bitter rot (caused by the fungus *Greeneria uvicola*), and Botryosphaeria rot (caused by *Botryosphaeria* spp.), which is commonly associated with trunk disease. Minor rots are caused by *Aspergillus* spp. (mainly black *Aspergilla*), *Cladosporium* spp., *Penicillium* spp., and *Rhizopus* spp. These bunch rots impact wine composition and quality through the production of compounds potentially responsible for off-flavors and aromas in wine or the production of mycotoxins, such as ochratoxin A and patulin (Battilani and Pietri, 2002; Barata et al., 2008a; Steel et al., 2013; Rousseaux et al., 2014). SR has recently become increasingly relevant (Brischetto et al., 2024).

Grape berries affected by SR show oxidation of the grape skin, which turns brown in red and white varieties and then becomes extremely fragile and cracks. The softening of the berry follows the disaggregation of the internal berry tissue (Gravot et al., 2001; Hall et al., 2018b). Rotten berries are characterized by a strong and pungent smell as the result of the production of several chemical compounds, such as acetic acid, glycerol, ethyl acetate, ethanol, galacturonic acid, acetaldehyde, and gluconic acid (Marchetti et al., 1984; Zoecklein et al., 2000). The etiology of SR is complex. Multiple microorganisms, including yeasts, bacteria, and filamentous fungi (Barata, 2011; Hewstone et al., 2007; Steel et al., 2013), have been isolated from affected berries, with high variability among the studies conducted in different years, regions, and viticultural contexts (Brischetto et al., 2024). SR depends on infestation by *Drosophila* spp. flies (Diptera: Drosophilidae) (Hall et al., 2018a). Adults of *D. melanogaster*, the vinegar fly, and other *Drosophila* spp. (Vercesi and Bisiach, 1987) deposit eggs onto exposed fruit pulp and larvae preventing the healing of wounds through their movements and favoring the penetration of SR-related microorganisms in the presence of lesions on the berry skin (Barata et al., 2012a; Fermaud et al., 2002). In contrast, *D. suzukii*, the spotted wing fly, can lay eggs in unwounded grapes (Atallah et al., 2014; Rombaut et al., 2017).

A recent systematic literature review (Brischetto et al., 2024) showed that there is still uncertainty about the microorganisms primarily involved in SR etiology and whether the microorganisms involved differ by region or vary in abundance between symptomatic and asymptomatic grape berries. Indeed, only 10 papers have focused on the differences between microbial communities associated with healthy and rotten grapes. Most of these papers were based on classical cultural techniques, which led to the misestimation of microbial communities (Lleixà et al., 2018). More recent molecular methods provide a better picture of microbial populations (Nocker et al., 2007) associated with plant diseases (Huang et al., 2017; Shen et al., 2018; Brady et al., 2017). Hall et al. (2019) used high-throughput sequencing to characterize the microbiome of SR-affected grapes in New York, US, and Tasmania, AUS. *Acetobacter* spp. were more abundant in rotten berries than in healthy ones. The yeast genera *Candida*, *Hanseniaspora*, *Pichia*, and *Saccharomyces* were abundant in healthy and rotten berries. However, SR-associated organisms were grouped primarily by location, not by presence/absence of symptoms or grapes. Gao et al. (2020) conducted a metagenomic analysis to determine the diversity and abundance of bacteria and fungi in spoiled table grapes collected in eastern coastal China. The dominant bacteria genera in SR-affected grapes were *Acetobacter*, *Gluconobacter*, *Bacillus*, and *Lactococcu*s. *Issatchenkia terricola*, *Colletotrichum viniferum*, *Hanseniaspora vineae*, *Saprochaete gigas*, and *Candida diversa* were dominant among fungi. Finding robust relationships between grape microflora and SR is relevant for extending the research to other grape-growing areas.

This study aimed to (i) determine the fungal and bacterial microbiota of healthy and rotten (i.e., exhibiting visual and olfactory SR symptoms) ripe bunches from different grape-growing areas of Italy over three years, (ii) characterize the diversity and composition of these microbiomes, and (iii) identify the microorganisms significantly associated with SR.

## 2 Materials and methods

### 2.1 Sample collection

Grape samples were collected from 39 vineyards in six Italian grape-growing regions (i.e., Veneto, Friuli Venezia Giulia, Emilia Romagna, Toscana, Lazio, and Puglia) in 2019, 2020, and 2021 (Table 1). In each vineyard, ripe bunches (BBCH 89; Lorenz et al., 1995) were harvested and divided into two categories: healthy (i.e., without any rot symptoms) and rotten (i.e., exhibiting visual and olfactory SR symptoms). Fifteen random bunches were collected for each category.

**Table 1:** The main characteristics of the vineyards considered in the present work.

Year	Grape variety		Sampling date	Village and region	Climate type^1^
2019	Crimson	Table	18/09	Turi (BA)	Cfa
2019	Italia	Table	17/09	Acquaviva (BA)	Cfa
2019	Italia	Table	17/09	Trinitapoli (BT)	Cfa
2019	Italia	Table	17/09	Casamassima (BA)	Cfa
2019	Riesling Renano	Wine	24/09	Prepotto (UD)	Cfb
2019	Regina-Pizzutello	Wine	18/09	Ruvo Di Puglia (BA)	Cfa
2019	Italia	Table	18/09	Trani (BT)	Cfa
2019	Vernaccia Di San Gimignano	Wine	30/09	Cenaia (PI)	Csa
2019	Montepulciano	Wine	25/09	San Severo (FG)	Cfa
2019	Corvina^*^	Wine	01/10	Verona (VR)	Cfa
2019	Chardonnay^*^	Wine	09/10	Marostica (VI)	Cfa
2019	Prosecco^*^	Wine	08/10	Fontanafredda (PN)	Cfb
2019	Malvasia Di Candia^*^	Wine	18/10	Frascati (RM)	Csa
2019	Pinot Bianco^*^	Wine	08/10	Guia (TV)	Cfa
2019	Cabernet Sauvignon^*^	Wine	09/10	Marostica (VI)	Cfa
2019	Merlot^*^	Wine	09/10	Marostica (VI)	Cfa
2019	Pinot Nero^*^	Wine	09/10	Marostica (VI)	Cfa
2019	Rondinella	Wine	08/10	San Quirino (PN)	Cfb
2019	Corvinone	Wine	09/10	Marostica (VI)	Cfa
2019	Corvina	Wine	08/10	Guia (TV)	Cfa
2019	Croatina	Wine	08/10	Fontanafredda (PN)	Cfb
2019	Malvasia Di Candia^*^	Wine	16/10	Roma (RM)	Csa
2019	Italia^*^	Table	17/10	Fiumicino (RM)	Csa
2020	Primitivo	Wine	31/08	San Ferdinando Di Puglia (BT)	Cfa
2020	Autumn Crisp Seedless	Table	31/08	Casamassima (BA)	Cfa
2020	Sangiovese	Wine	21/09	Cenaia (PI)	Csa
2020	Moscato Giallo	Wine	02/10	Castell’Arquato (PC)	Cfa
2020	Glera	Wine	14/09	Conegliano (TV)	Cfb
2020	Cabernet Franc	Wine	05/10	Calamsino – Bardolino (VR)	Cfa
2020	Cabernet Sauvignon	Wine	05/10	Marostica (VI)	Cfa
2020	Chardonnay	Wine	25/09	Marostica (VI)	Cfa
2021	Fiammetta	Wine	27/09	Turi (BA)	Cfa
2021	Vernaccia Di San Gimignano	Wine	27/09	Cenaia (PI)	Csa
2021	Fleurtai (PIWI)^2^	Wine	30/09	Piacenza (PC)	Cfa
2021	Solaris (PIWI)	Wine	30/09	Piacenza (PC)	Cfa
2021	Felicia (PIWI)	Wine	30/09	Piacenza (PC)	Cfa
2021	Johanniter (PIWI)	Wine	30/09	Piacenza (PC)	Cfa
2021	Calardis Blanc (PIWI)	Wine	30/09	Piacenza (PC)	Cfa
2021	Rkatsiteli (PIWI)	Wine	30/09	Piacenza (PC)	Cfa

In each vineyard, 15 healthy and 15 sour-rot rotten bunches were sampled close to harvest. ^*^No healthy bunches were found. ^1^Köppen-Geiger climate classification and the acronyms represent. Cfa, Humid Subtropical Climate; Cfb, Oceanic Climate; Csa, Hot-Summer Mediterranean Climate (Peel et al., 2007). ^2^PIWI: grapevine varieties showing resistance to downy/powdery mildews.

For each sample, 100 berries were randomly removed from the bunches with sterilized scissors, placed in a plastic bag, and manually pressed. Then, 100 mL of the obtained must (i.e., a blend of pulp and juice obtained from the crushing of the berries) was extracted and placed into two 50 mL Falcon tubes. The samples were stored at −20°C until molecular analysis was performed.

### 2.2 DNA extraction, amplification, and sequencing

Must samples were sent to WineSeq laboratories^1^, ^2^ for total DNA extraction and high-throughput sequencing. Samples were processed using the Qiagen PowerSoil® DNA isolation Kit and analyzed for the 16 s rRNA V4 region and the ITS by amplification of the ITS1 region using WineSeq® custom primers (Patent WO2017096385). After quality control by electrophoresis gel, each library (16S and ITS) was pooled in equimolar amounts and subsequently sequenced on an Illumina MiSeq instrument (Illumina, San Diego, CA, USA) using 2×301 paired-end reads and according to the Biome-Makers implemented protocol. All the data produced and collected were subsequently analyzed using a QIIME-based custom bioinformatics pipeline (Patent WO2017096385, Biome Makers). The first quality control was used to remove adapters and chimeras. Later, the readings were trimmed, and sequence variant (SV) clusters were performed using 97% identity. SV clusters were compared with the WineSeq® taxonomy database (Patent WO2017096385) to identify the entire microbial population (bacteria, yeasts, and filamentous fungi) (Belda et al., 2017).

### 2.3 Data analysis

The fungal and bacterial SVs shared among bunch status were obtained using a Venn diagram analysis using the software available at: http://bioinformatics.psb.ugent.be/, accessed in June 2023.

Data analyses were performed using MicrobiomeAnalyst (Dhariwal et al., 2017; Chong et al., 2020). Data were filtered before the analysis based on the following criteria to remove low-quality or uninformative features: (i) SVs with less than four reads in a minimum of 20% of samples, and (ii) SVs with less than 10% inter-quantile range were excluded, because very small counts in very few samples are likely due to sequencing errors or low-level contaminations, and those that are close to constant throughout the experiment conditions are unlikely to be associated with the conditions under study.

Alpha diversity was calculated using Shannon and Chao1 indices in the Phyloseq package, and beta diversity was estimated using a principal coordinates analysis (PCoA) based on Bray–Curtis metrics (Vázquez-Baeza et al., 2013) with MicrobiomeAnalyst. PERMANOVA analysis was used to evaluate which SVs significantly differed in abundance among the experimental factors.

The linear discriminant analysis effect size (LEfSe) algorithm was used to identify taxa at the genus level that differed in relative abundance between bunch status (healthy and rotten) (Segata et al., 2011). MicrobiomeAnalyst LEfSe implementation was used. The threshold for the logarithmic linear discriminant analysis (LDA) score was set at 2.0, and the FDR-adjusted *p*-value cutoff was set at 0.1.

A correlation network analysis was performed using MicrobiomeAnalyst based on the SparCC algorithm (Friedman and Alm, 2012). The permutation was settled at 100, with a *p*-value threshold of 0.01 and a correlation threshold of 0.5 at the genus taxonomic level.

## 3 Results

**Figure 1:**
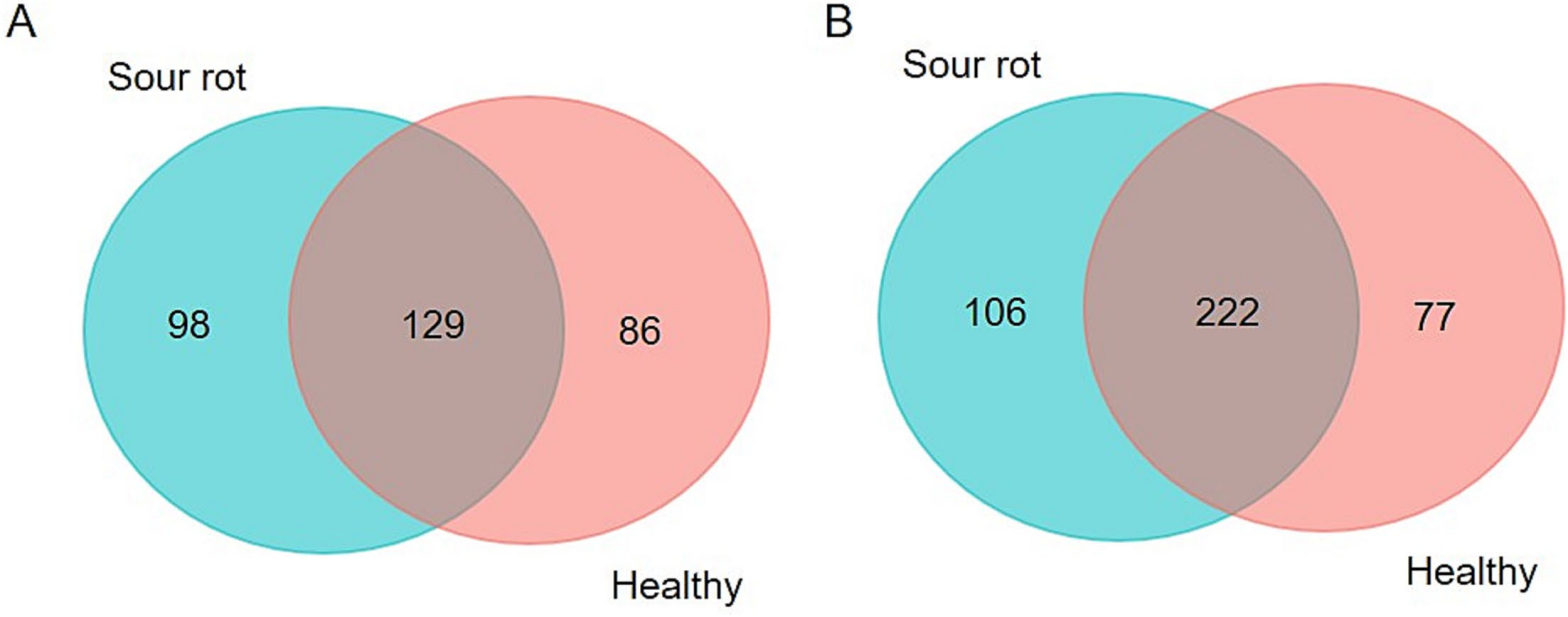
Venn diagram illustrating the overlap of the number of SVs identified in the fungal (A) and bacterial (B) microbiota between grapevine bunches showing sour rot symptoms or not (referred to as healthy).

A total of 313 fungal SVs were annotated in our study for a total of 8,260,221 reads; 41.2% of these SVs were present in both healthy and rotten bunches, and 31.3% were associated with rotten bunches only (Figure 1A). A total of 405 bacterial SVs were annotated for 3,468,723 reads; 26.2 and 19.1% of bacterial SVs were associated with rotten and healthy bunches, respectively, while the majority of bacterial SVs (54.7%) were in common (Figure 1B).

For robust statistical analysis, 146 fungal SVs were eliminated for low abundance and four for low variance. Therefore, the analysis was conducted on 27 SVs. For bacterial SVs, 219 were eliminated for low abundance and seven for low variance, so 59 SVs (2,852,372 reads) were used. The numbers of reads for all SVs are shown in the Supplementary Table S1.

**Figure 2:**
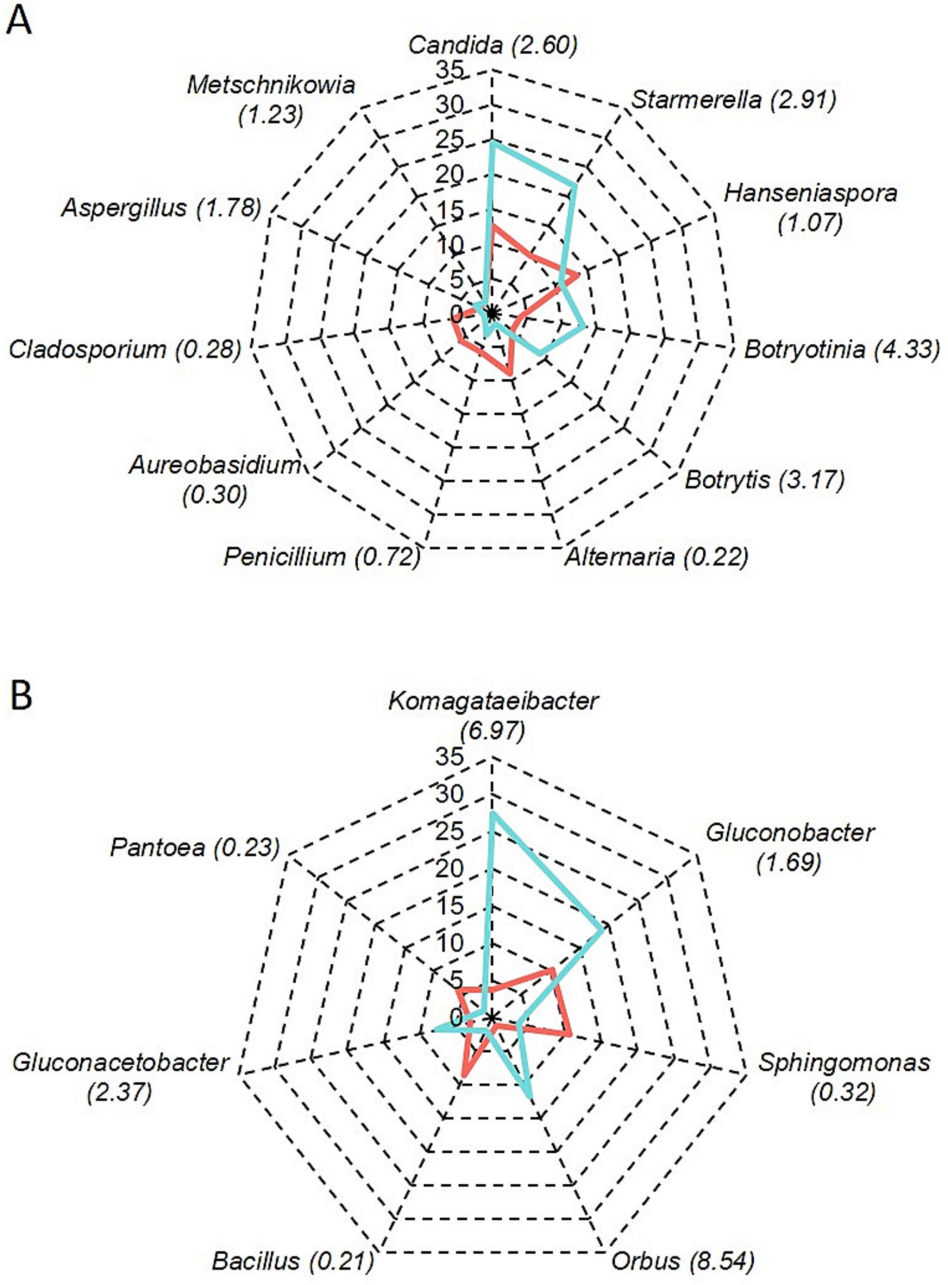
Relative abundance of fungal (A) and bacterial (B) genera in grapevine bunches showing sour rot symptoms (light blue line) or not (red line); the number between brackets shows the ratio of the number of reads in rotten vs. healthy bunches.

Figure 2 shows the relative abundance of fungal and bacterial genera detected in healthy and rotten bunches. The most abundant genera in healthy bunches were *Hanseniaspora* (13.4%), *Candida* (12.6%), *Starmerella* (9.9%), and *Alternaria* (9%) (Figure 2A). In rotten bunches, *Candida* was the most abundant genus (24.6%), followed by *Starmerella* (21.8%), *Botryotinia* (13.2%), *Hanseniaspora* (10.8%), and *Botrytis* (8.9%) (Figure 2A). *Sphingomonas* (10.6%), *Gluconobacter* (10.3%), and *Bacillus* (8.65%) were the most abundant genera in the healthy bunches (Figure 2B). The abundance of *Gluconobacter* spp. increased in rotten bunches (18.8%), while that of *Sphingomonas* spp. and *Bacillus* spp. decreased (3.6 and 2%, respectively). *Komagataeibacter* spp. (27.4%), *Orbus* spp. (11.8%), and *Gluconacetobacter* spp. (7.7%) were also abundant in rotten bunches. *Pantoea* spp. were present in healthy and rotten bunches with lower abundance (5.9 and 1.5%, respectively) (Figure 2B).

**Figure 3:**
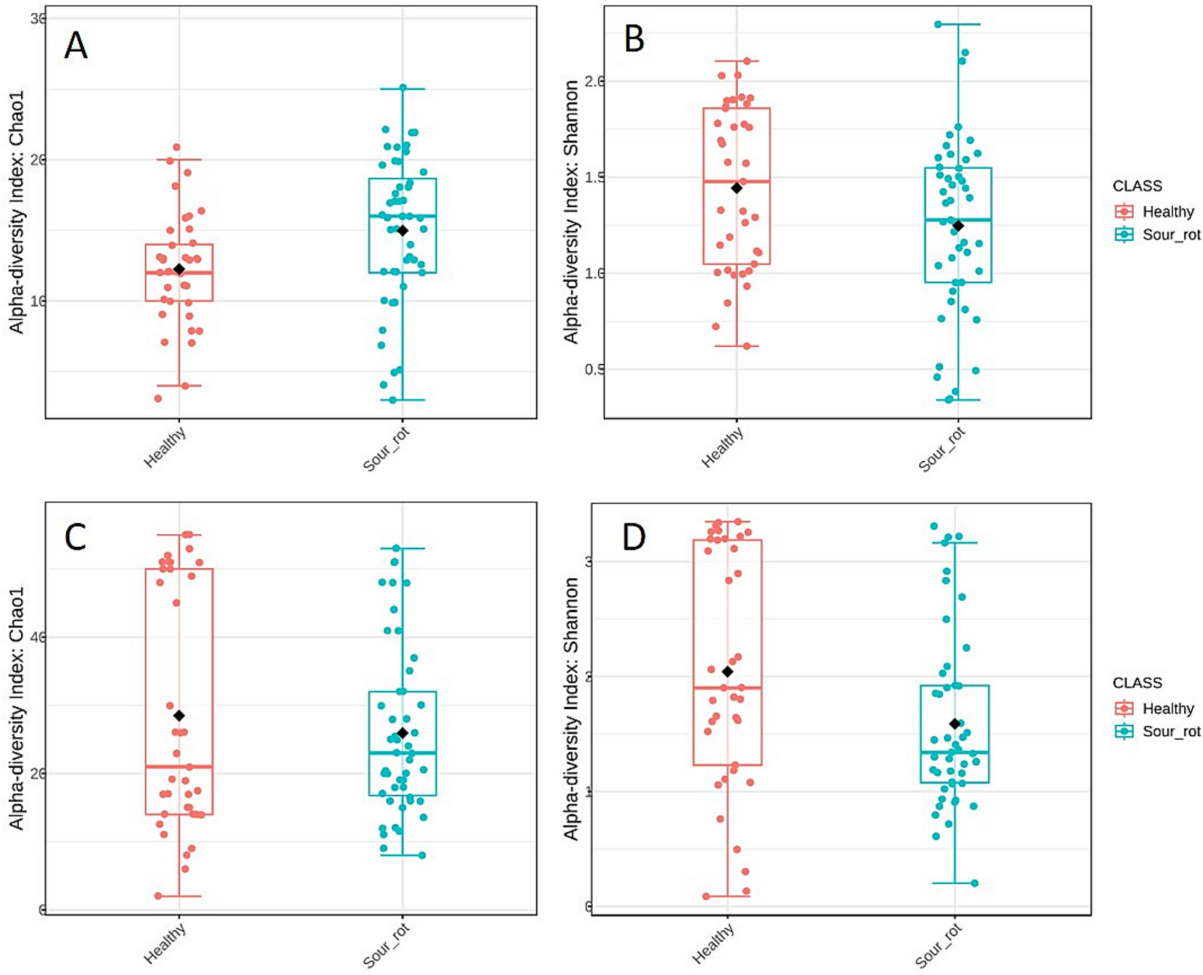
Boxplot illustrating the differences in the fungal (A,B) and bacterial (C,D) communities in healthy (red) and rotten (light blue) bunches based on Chao1 (A,C) and Shannon (B,D) diversity indicators. The box extends from the 25th to the 75th quartile of the data distribution, the line crossing the box represents the median, and the black diamond indicates the average; whiskers extend to the maximum and minimum.

The alpha diversity of fungal communities, which reflects the distribution of SV abundances in a single sample, differed among the samples. Chao1, which is based on the abundance of SVs belonging to a genus in a sample, and Shannon, which accounts for both the number of SVs and their relative abundance, both indicated that fungal diversity in healthy bunches was significantly different from that in rotten bunches, with *p* = 0.008 and 0.047, respectively (Figures 3A,B). The alpha diversity of bacterial communities was significantly different in healthy and rotten bunches only for the Shannon estimator (Chao1: *p* = 0.460; Shannon: *p* = 0.030) (Figures 3C,D). These results indicated that healthy and rotten bunches differed in the total number of species, but not in abundance distribution across species.

**Figure 4:**
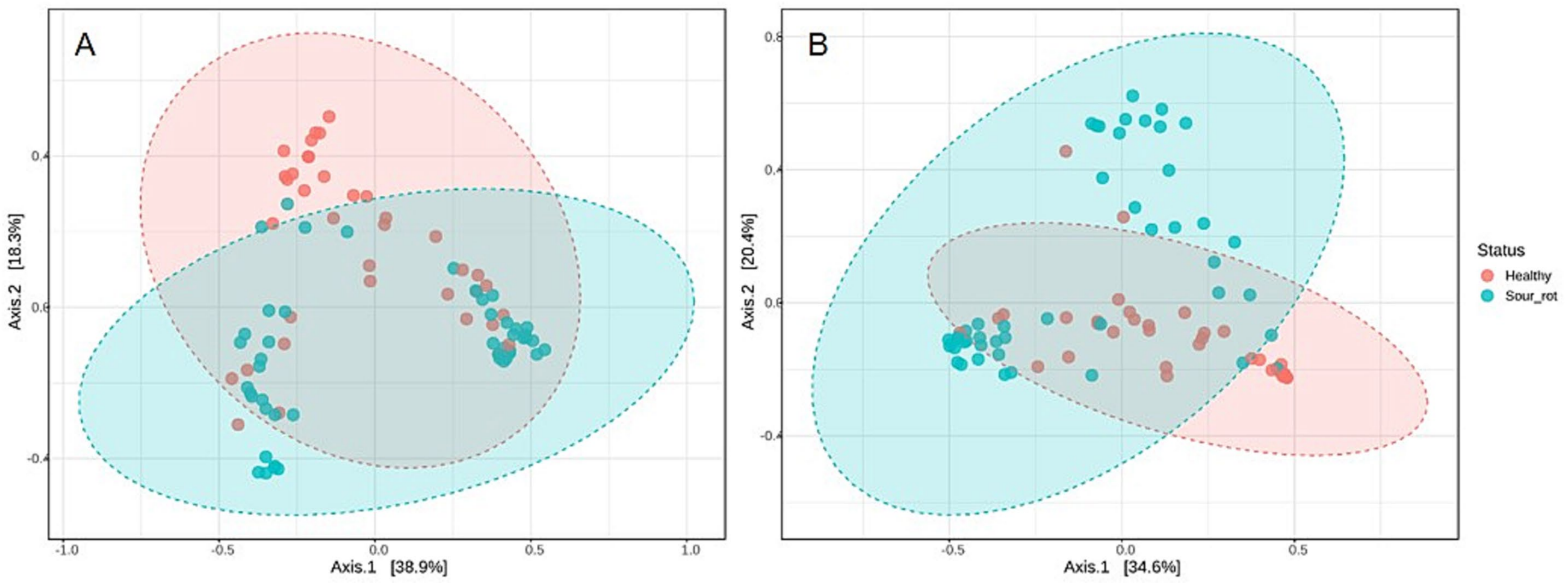
Principal coordinate analysis (PCoA) based on Bray–Curtis dissimilarity metrics, showing the distance in the fungal (A) and bacterial (B) communities present in healthy (red dots) and rotten (light blue dots) bunches. Areas show distinct clustering of healthy (red) and rotten (light blue) bunches.

The beta diversity of fungal and bacterial communities, which focuses on SV dissimilarities between samples, measured with the Bray–Curtis dissimilarity, is presented using the PCoA in Figure 4. The bunch status (healthy or rotten) was a significant source of beta diversity for both fungal (R^2^ = 0.074, *p* = 0.002; Figure 4A) and bacterial (R^2^ = 0.012, *p* = 0.001; Figure 4B) communities. This result indicated that the microbiome composition differed in healthy and rotten bunches.

**Figure 5:**
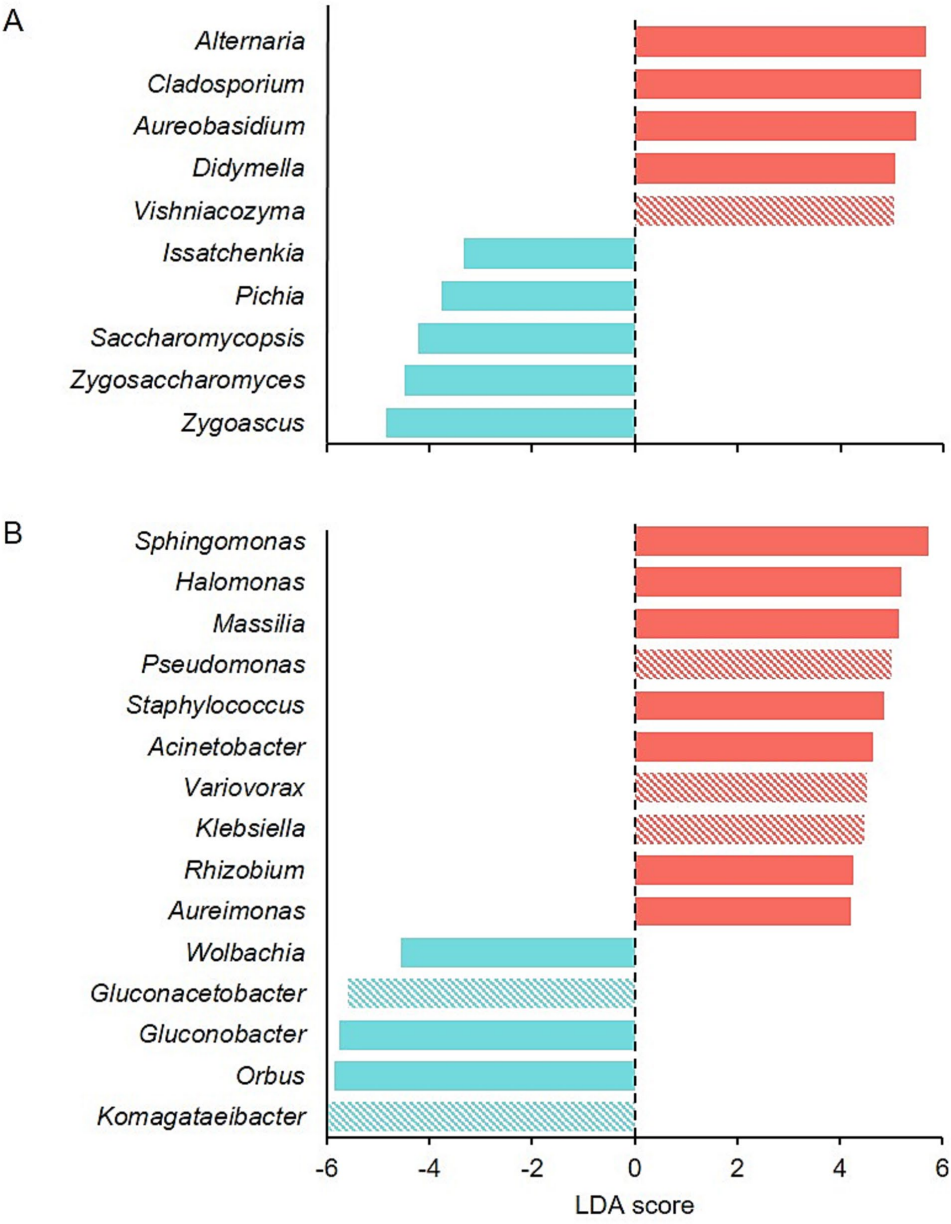
Graphical summary of LEfSe analysis for fungal (A) and bacterial (B) communities in healthy (red) and rotten bunches (light blue). The LDA score represents the extent to which the genera differ among the groups: the higher the positive score, the higher the increase in the relative abundance of the genus concerning rotten bunches, and the lower the negative score, the higher the increase in the relative abundance of the genus in rotten concerning healthy bunches. Full and diagonally striped bar colors mean *p*-values <0.01 and <0.05, respectively.

LEfSe detected 10 fungal genera as the main determinants of the dissimilarities between healthy and rotten bunches (Figure 5A). *Zygosaccharomyces* (*p* ≤ 0.001), *Zygoascus* (*p* < 0.001), *Saccharomycopsis* (*p* = 0.004), *Issatchenkia* (*p* = 0.004), and *Pichia* (*p* = 0.002) were the most important fungal genera that distinguished rotten bunches, with LDA scores between −3.33 and − 4.85 (Figure 5A), even though they accounted for approximately 3.5% of the total reads in rotten bunches. Fifteen bacterial genera differed between healthy and rotten bunches, which specifically were *Orbus* (*p* < 0.001), *Gluconobacter* (*p* < 0.001), and *Wolbachia* (*p* = 0.004), and with lower significance, *Komagataeibacter* (*p* = 0.030) and *Gluconacetobacter* (*p* = 0.011), showing an LDA score < −6. Therefore, the rotten bunches had statistically consistent differences (Figure 5B).

**Figure 6:**
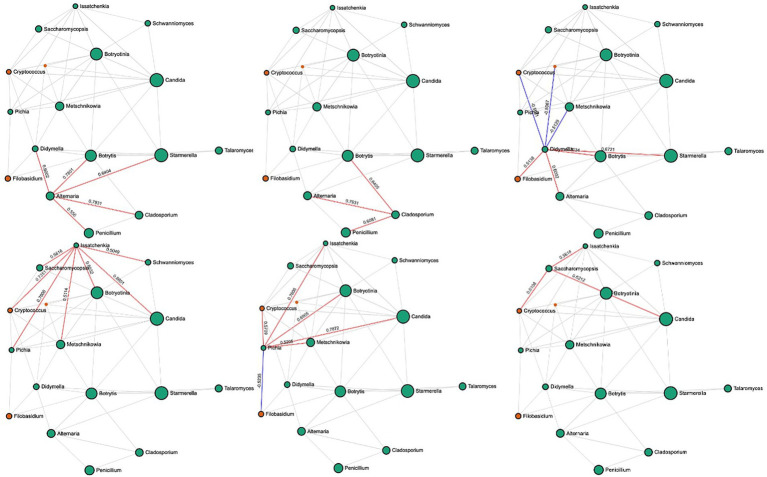
SparCC correlation analysis for fungal communities in rotten bunches. Nodes represent taxa at the genus level. Node size is based on the number of connections to each taxon. Edges represent correlations between pairs: red and blue edges represent positive and negative correlations, respectively; the value is the correlation coefficient between taxa. The nodes are colored based on phyla: green for Ascomycota and orange for Basidiomycota.

**Figure 7:**
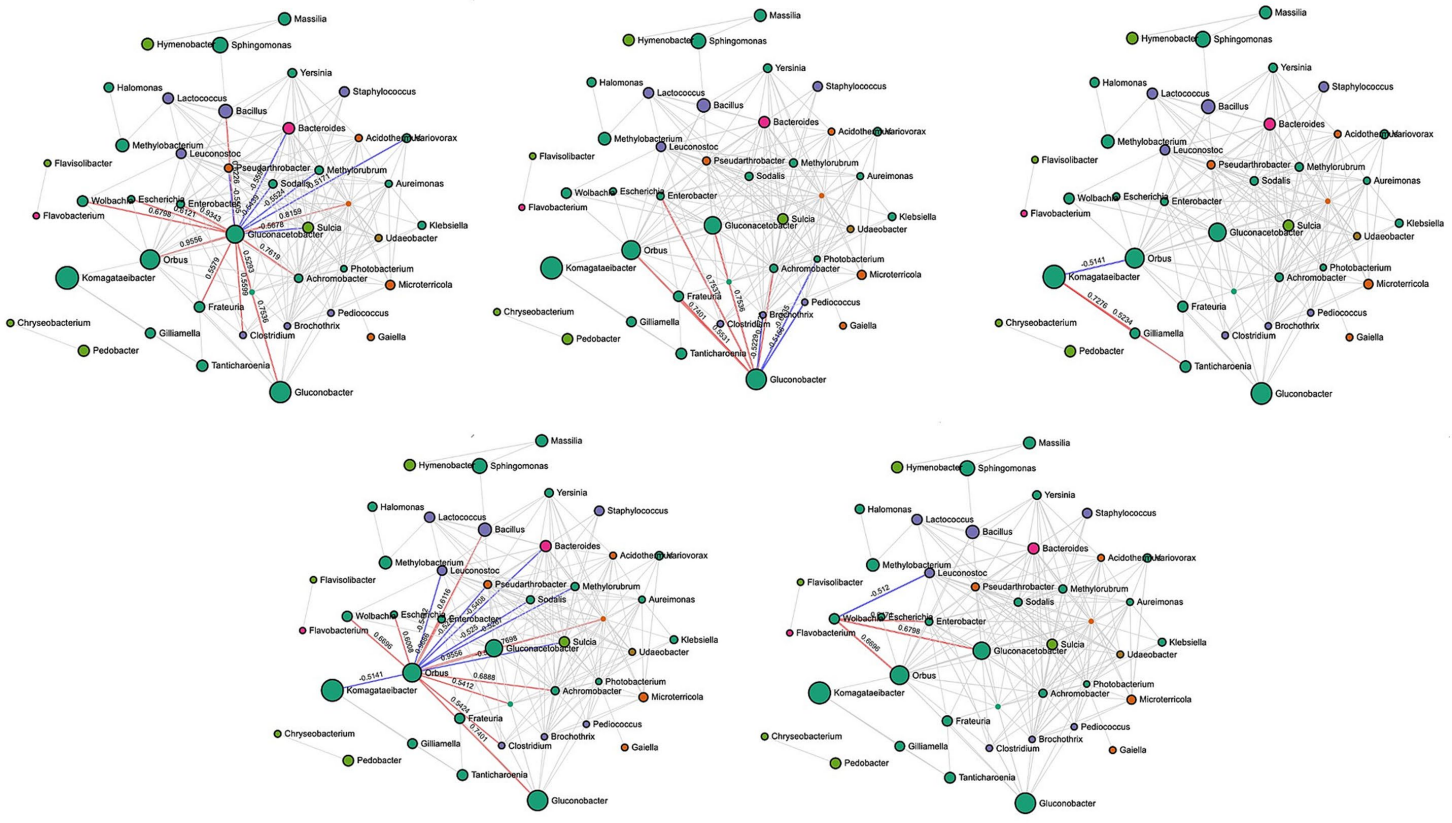
SparCC correlation analysis for bacterial communities in rotten bunches. Nodes represent taxa at the genus level. Node size is based on the number of connections to each taxon. Edges representing correlations between pairs: red and blue edges represent positive and negative correlations, respectively; the value is the correlation coefficient between taxa. The nodes are colored based on phyla: green for Proteobacteria, orange for Actinobacteria, purple for Firmicutes, pink for Bacteroidetes, green for Gemmatimonadota, yellow for Bacteroidota, and brown for Verrucomicrobiota.

In rotten bunches, 96 and 383 significant edges and connections were observed through the correlation network analysis between the fungal (Figure 6) and bacterial (Figure 7) genera, respectively. Positive correlations indicated that genera are likely to coexist, while negatively related genera competitively exclude each other. In particular, the most important fungal genera that characterized rotten bunches, such as *Issatchenkia* and *Pichia,* correlated positively among them (0.761) and *Candida* (0.869 and 0.787, respectively), which was the most abundant genus in rotten bunches. Concerning the bacteria, the genera strongly associated with bunches showing SR symptoms, such as *Gluconacetobacter, Orbus*, and *Wolbachia,* were positively correlated with each other, with the strongest correlation between *Gluconacetobacter* and *Orbus* (0.956). However, the latter was negatively correlated with *Komagataeibacter* (−0.514), one of the most abundant genera characterizing rotten bunches.

## 4 Discussion

Our study analyzed the fungal and bacterial microbiomes of grape bunches affected by SR to determine the main differences from healthy bunches. Information about the microorganisms more likely to be associated with the disease was also inferred.

Grape berries host a complex microbial community comprising bacteria, yeasts, and filamentous fungi (Barata et al., 2012b; Rousseaux et al., 2014; Fleet, 2003; Ribéreau-Gayon et al., 2006), which play a prominent role in the winemaking process and wine quality (Barbe et al., 2001; Nisiotou et al., 2011; Verginer et al., 2010; Pretorius, 2000). Some yeasts can benefit winemaking, while others can negatively affect wine quality (Martins et al., 2014). Other microorganisms are considered spoilage agents, such as filamentous fungi, which may influence the safety or sensory quality of wines (Barata et al., 2011; Rousseaux et al., 2014; Steel et al., 2013; Barbe et al., 2001; Nisiotou et al., 2011; Verginer et al., 2010). Similarly, some bacteria participate in wine fermentation, such as lactic acid bacteria (LAB), which conduct malolactic fermentation, improving wine flavor and stability (Lonvaud-Funel, 1999). Other bacteria, such as *Acetobacter* spp., are detrimental to wine quality because of acetic acid production (Barata et al., 2012b).

Our analysis showed that the number of fungal and bacterial SVs was higher in SR-affected than in healthy bunches. This result agrees with the literature review by Brischetto et al. (2024), which revealed that 69 and 128 microorganisms were isolated from unaffected and affected berries, respectively. Thirty different genera were found in the previous literature on SR (Brischetto et al., 2024), and 28 were found in our samples, even though there was no complete agreement about whether they were associated with healthy or rotten bunches (Supplementary Figure S1). For example, *Botrytis* spp. were previously found occasionally in rotten bunches only. Still, this genus and its teleomorph, *Botryotinia*, accounted for 22.7% of reads in rotten bunches and 7.8% in healthy ones (Supplementary Figure S1A). Of the 30 bacterial genera mentioned in previous literature on SR (Brischetto et al., 2024), 25 (86.2%) were also found in our samples, and their presence in healthy/rotten bunches was not always in agreement with the literature (Supplementary Figure S1B). For instance, *Bacillus* spp. was reported to be prevalent in rotten bunches (Brischetto et al., 2024), but it was more abundant in healthy (8.7% of the total reads) than in rotten (2%) bunches in our samples.

Our microbiome analysis revealed that the richness and evenness of both species (as shown by significant alpha diversity estimators) were influenced by bunch status, and the microbiome composition significantly varied between healthy and rotten bunches (as demonstrated by a significant beta diversity indicator). Overall, the microbial profile of rotten bunches was characterized by the yeast genera *Zygosaccharomyces*, *Zygoascus, Saccharomycopsis, Issatchenkia*, and *Pichia* and by the bacterial genera *Orbus*, *Gluconobacter*, *Wolbachia*, *Komagataeibacter*, and *Gluconacetobacter*, which frequently coexisted, being closely correlated with each other. The contemporary presence of yeasts and bacteria, especially the so-called acetic acid bacteria (AAB), in grape berries showing typical SR symptoms has been previously documented (see Brischetto et al., 2024). Hall et al. (2018a) postulated a succession of these microorganisms during disease development, with yeasts producing ethanol from sugars and AAB using ethanol to produce acetic acid.

Our analysis showed that *Zygosaccharomyces*, *Zygoascus*, *Saccharomycopsis, Issatchenkia*, and *Pichia* were characteristic of SR-affected bunches, even though they accounted for less than 3.5% of the total reads. These yeast genera belong to the order Saccharomycetales, families Saccharomycetaceae, Trichomonascaceae, Saccharomycopsidaceae, and Pichiaceae, respectively, which globally accounted for 58.3% of the total reads in our SR-affected samples.

Several species in the yeast genus *Zygosaccharomyces* are well-known spoilage microorganisms for their high sugar, ethanol, and acetic acid tolerance (Palma et al., 2018; James and Stratford, 2003). These microorganisms are considered rare contaminants of grapes but are among the most dangerous wine spoilers (Fleet et al., 2002; Barata et al., 2008a). Fermentative species belonging to *Zygoascus* have been characterized as producers of biogenic amines in wine (Tristezza et al., 2013). In particular, *Z. hellenicus* (teleomorph of *Candida steatolytica*) has been described as a contaminant often associated with damaged grapes (Barata et al., 2008b). Some *Saccharomycopsis* (specifically, *S. vini* and *S. crataegensis*) have been previously associated with SR (Barata et al., 2008a, 2008b; Bisiach et al., 2021; Guerzoni and Marchetti, 1982; Marchetti et al., 1984), with *S. crataegensis* being characterized by lipolytic activity and reproducing the disease symptoms when inoculated in combination with other microorganisms, such as *Issatchenkia occidentalis* and *Kloeckera apiculata* (Guerzoni and Marchetti, 1987).

*Pichia* spp. are yeasts that consume glucose without ethanol formation (Varela and Varela, 2019; Vicente et al., 2021), and *Issatchenkia* spp. can ferment glucose to ethanol in acidic media (Hisamatsu et al., 2006). These genera are closely related, and some species of *Issatchenkia* have been proposed to be classified within *Pichia* (Kurtzman et al., 2008). All of these yeasts have been previously isolated from SR-affected bunches (Guerzoni and Marchetti, 1987; Fleet et al., 2002; Nisiotou and Nychas, 2007; Barata et al., 2008a, 2008b, 2012a), and the yeasts *Zygoascus hellenicus* and *Issatchenkia* spp. have been proposed as biomarkers for SR (Barata et al., 2012b).

The yeasts are also part of the *D. melanogaster* microbiome (Broderick and Lemaitre, 2012), with *Hanseniaspora*, *Candida*, *Zygoascus, Saccharomycopsis,* and *Pichia* being commonly associated with natural *Drosophila* spp. populations (Chandler et al., 2012; Stamps et al., 2012; Hamby et al., 2012; Scheidler et al., 2015; Begon, 1982; Ganter, 2006; Barata et al., 2012a). The interactions between *Drosophila* spp. and yeasts appear mutualistic, as yeasts affect several aspects of insect physiology, behavior, and immunity (Hoang et al., 2015). Ingestion by the larvae of some yeasts speeds up larval development time and increases adult body weight (Anagnostou et al., 2010). Yeasts can survive digestion by *D. melanogaster*, so flies serve as yeast vectors under natural conditions (Reuter et al., 2007; Coluccio et al., 2008).

In our samples, bacteria of the family Acetobacteraceae accounted for 56.5% of total bacterial reads in SR-affected bunches, with the genera *Komagataeibacter, Gluconacetobacter,* and *Gluconobacter* representing 53.8% of these reads. *Komagataeibacter* was the most abundant genus found in our samples (27.4% of the total reads in rotten bunches). This genus was recently defined to include several species previously classified as *Gluconacetabacter* (Yamada et al., 2012; Mateo et al., 2014). These bacteria have been isolated from rotten grape bunches (Mateo et al., 2014; Gopu and Govindan, 2018; Gao et al., 2020; Srivastava and Mathur, 2022; Ryngajłło et al., 2020) and can produce gluconic acid from glucose and other sugars, and oxidate ethanol to acetic acid (Gomes et al., 2021). *Komagataeibacter* spp. is also an efficient bacterial cellulose producer from various carbon and nitrogen sources (Islam et al., 2017), including grape pomace (Gorgieva et al., 2023). *Gluconacetobacter* spp. and *Gluconobacter* spp. were among the most prevalent bacteria in the affected berries in previous studies (Brischetto et al., 2024). Together with *Acetobacter* spp., they have been associated with grape and wine spoilage (Joyeux et al., 1984; Navarro et al., 2013; Hall et al., 2019). Species of *Acetobacter* are often isolated from wine due to their ethanol tolerance, whereas *Gluconobacter* spp. prefer sugar-rich environments with low amounts of alcohol (Campaniello and Sinigaglia, 2017). *Gluconobacter* spp. oxidize grape sugars primarily using gluconic acid (Batt and Tortorello, 2014). Some *Gluconacetobacter* spp. produce thick leathery pellicles in the air liquid during winemaking, which is considered a contaminant (Rani et al., 2011). Unlike previous studies (Hall et al., 2019), *Acetobacter* spp. had a low abundance in our samples (1.76% of total fungal reads).

In addition to yeasts, Acetobacteraceae has been frequently associated with *Drosophila* spp. in nature (Staubach et al., 2013). *Gluconobacter* spp. was the most prevalent bacteria in wild-caught flies in some studies (Staubach et al., 2013; Corby-Harris et al., 2007; Ryu et al., 2008), and present, but not prevalent in others (Chandler et al., 2011). *Komagataeibacter* spp. have also been found in the *Drosophila* spp. gut as a valuable microbiota member in overcoming environmental stress (Beribaka et al., 2021). The relationship between AAB, *Drosophila* spp., and SR has been demonstrated by Barata et al. (2012a). These authors did not observe SR when bunches inoculated with AAB were physically separated from insects, even when berries were artificially injured, because wounds in berry skin healed in the absence of *Drosophila* spp., thus preventing SR development. The authors then concluded that, in the vineyard, the induction of SR depends on the contamination of wounded berries by a microbial consortium transported by *Drosophila* spp. that act as vectors for microorganisms associated with grape SR. Hall et al. (2018a) postulated that the role of *Drosophila* spp. go beyond vectoring because, in artificial inoculation studies, SR symptoms developed only in the presence of *D. melanogaster*, either wild type or axenic. Softening of the berry pulp by the enzymes released by larvae to facilitate consumption (Gregg et al., 1990) may be an aspect to be considered. However, the AAB–*Drosophila* spp.–SR relationship seems even more complex.

AAB are considered ubiquitous symbionts of *Drosophila* spp. (Rosenberg et al., 2007; Douglas, 2018). AAB and other microorganisms are part of the microbial community within the intestine of *D. melanogaster* (Ryu et al., 2008). AAB and LAB metabolize ethanol and acetic acid within the gut of *D. melanogaster*, and the secondary metabolites produced are beneficial for the growth and development of the insect, both directly (Douglas, 2017; Fischer et al., 2017) and indirectly (Keebaugh et al., 2018). AAB and other bacteria are also transported in bristled areas or tarsal segments on the fly surface (Barata et al., 2012a; Hong et al., 2022), forming biofilms (Ren et al., 2007) and promoting the dispersal and establishment of these bacteria in fruit (Barata et al., 2012a). The fly surface microbiota is complex, and the bacterial richness of surface microbiomes is much higher than that of gut microbiota. Such microbiota may defend insects against fungal and parasitic infections (e.g., *Beauveria brassiana* and *Metarhizium robertsii*), inhibiting spore germination (Hong et al., 2022).

The presence of AAB in the substrate has beneficial effects on *D. melanogaster* larval growth and development time (Shin et al., 2011). Indeed, these bacteria are ingested by insects and become part of their gut microbiota (Wong et al., 2011), playing a significant functional role in the life of the host, including innate immunity (Ryu et al., 2008), lifespan (Clark et al., 2015; Lee et al., 2019), nutrition (Beribaka et al., 2021), reproduction (Leitão-Gonçalves et al., 2017), and behavior (Silva et al., 2021). Rosenberg et al. (2007) speculated that the microbial community associated with *Drosophila* spp. can be seen as an external organ of the fly holobiont. It is also known that bacteria are attractive to Diptera because of the production of a range of volatile compounds, including ammonia (Bateman and Morton, 1981; Robacker et al., 1998; MacCollum, 1992; Lauzon et al., 1998; Robacker and Lauzon, 2002). *Gluconobacter* spp. and *Komagataeibacter* spp. rapidly produced acetic acid and ethanol, which are attractive to *Drosophila* spp. (West, 1961; Landolt et al., 2012; Mazzetto et al., 2016). The volatile compounds produced by mutualistic microorganisms living inside host insects with a symbiotic relationship with plants trigger their trophic interaction (Frago et al., 2012) and strengthen the insect–bacteria relationship.

The genus *Orbus* represented 11.8% of the reads in SR-affected bunches. This genus was not previously reported as being associated with SR-affected berries (Brischetto et al., 2024). It was initially classified as Enterobacteriaceae, a large family that includes many animal- and plant-associated bacteria. It was reclassified into the family Orbaceae (order Orbales) within *γ*-Proteobacteria (Kwong and Moran, 2013). These bacteria were found to be free-living associates of many insects, including the gut of *D. melanogaster* (Cox and Gilmore, 2007), with several lineages being endosymbiotic and required for insect nutrition, defense from parasites, and tolerance of heat stress (Douglas, 1998; Montllor et al., 2002; Moran et al., 2005). Chandler et al. (2011) designated this entire lineage as “Enterobacteriaceae Group Orbus” and found it abundantly in *Drosophila* spp. samples, representing over 21% of all bacteria in natural *Drosophila* spp. populations. Other Enterobacteriaceae, such as *Enterobacter/Pantoea* and *Klebsiella* (present in our SR-affected samples with 1.9% abundance), participate in nitrogen cycling within the dipteran gut and serve as important contributors to insect survival in nature (Lauzon et al., 2000).

The genus *Wolbachia* represented 0.6% of the reads in SR-affected bunches. *Wolbachia* spp. bacteria are *α*-Proteobacteria and obligate endosymbionts that are extremely widespread in approximately 40% of all insect species and cause various types of reproductive phenotypes that favor vertical transmission and spread in populations (Werren et al., 2008; Fast et al., 2011). In *Drosophila* spp., *Wolbachia* spp. can have both deleterious and beneficial effects on different fitness components, such as fecundity, lifespan, and stress tolerance (Serga et al., 2021). Beneficial effects include improved reproduction related to a shortened lifespan and lower stress resistance (Serga et al., 2021). The presence of *Wolbachia* spp. changes the composition of the bacterial communities in the *Drosophila* spp. fly gut, decreasing its biodiversity, particularly by reducing the abundance of *Acetobacter* spp. (Yixin et al., 2017). It may be speculated that the presence of *Orbus* and *Wolbachia* genera in SR-affected berries and other Enterobacteriaceae of the *Drosophila* spp. gut microbiota is related to the presence of *Drosophila* spp. in those berries.

**Figure 8:**
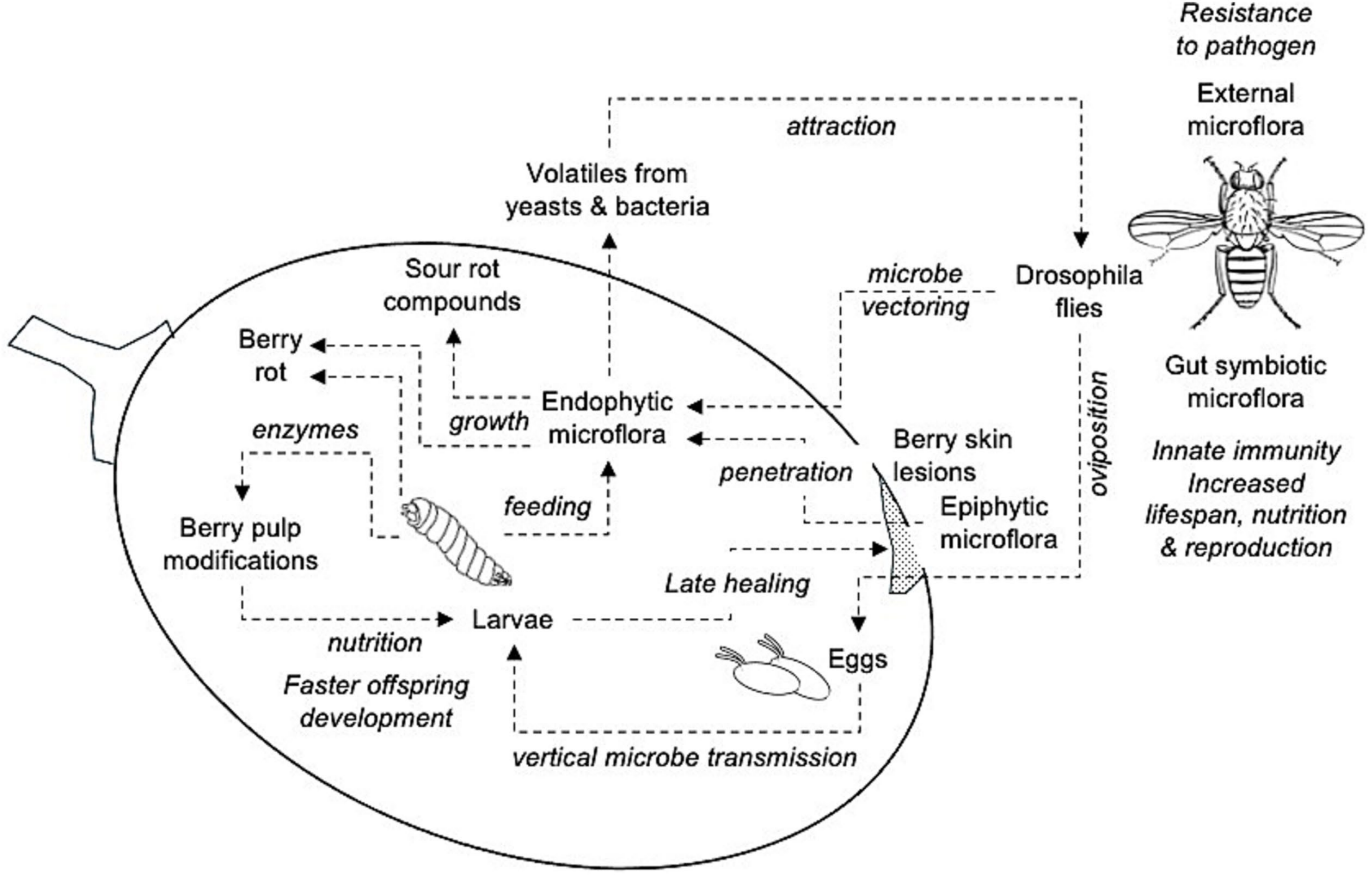
Schematic relationship of the interactions between yeasts and bacteria associated with grape sour rot and *Drosophila* spp., as from literature. These relationships agree with the microbial composition of the SR-affected bunches in this study. Flying adults carry microorganisms on both external parts of their bodies and in the gut as symbionts, which provide multiple benefits to insects. Adults deposit eggs in the berry pulp through berry skin lesions (e.g., *D. melanogaster*) or directly (e.g., *D. suzukii*) so that the epiphytic yeasts and bacteria can penetrate the pulp or enter the pulp through vectoring on fly body parts or vertical transmission (adults to eggs). Larvae develop into the berry feeding the endophytic microorganisms and berry pulp components through modifications induced by insect-released enzymes; this results in faster offspring development. Larvae also delay wound healing through movement. Endophytic microorganisms grow and produce berry rot and the compounds associated with sour rot (ethanol, acetic acid, gluconic acid, etc.). Some of these compounds are volatiles that attract flies.

In conclusion, our analysis revealed that even if approximately 570 microorganisms were found in grape bunches affected by SR collected in 39 vineyards in six Italian grape-growing regions characterized by different pedoclimatic conditions over three years, few bacteria and yeast were closely related to the presence of the disease. These microorganisms were also found in previous studies, so we can consider our results sufficiently robust. All these microorganisms have been previously associated with wild *Drosophila* spp. in the literature, with a complex relationship that can be depicted in Figure 8.

Further studies on the association between SR microorganisms and *Drosophila* spp. could contribute to explaining the differences in the microflora composition and abundance between different studies. Indeed, the gut microbiota composition of *Drosophila* species varies in association with diet, genotype, laboratory, and age (Broderick and Lemaitre, 2012; Chandler et al., 2011; Staubach et al., 2013; Clark and Walker, 2018; Marra et al., 2021). For instance, the microbial diversity in the guts of *D. suzukii* differs from that of *D. melanogaster* because of the adaptation of the former to its high-sugar ecological niche (Lin et al., 2021). A better understanding of the SR–*Drosophila* spp. relationships could also open up perspectives for disease control. The literature review by Brischetto et al. (2024) showed that disease control based on using fungicides, natural products, and biocontrol microorganisms, either alone or in an integrated pest management strategy, provided inconsistent, often poor, control. An indirect SR control targeted at flies has been proposed by Bisiach et al. (2021). In some viticultural areas of the US, fly control was achieved by using various insecticides (Weigle et al., 2020), primarily pyrethroid zeta-cypermethrin, which however, has been associated with increased fly resistance to insecticides (Sun et al., 2019; Mertz et al., 2022, 2023; Hubhachen et al., 2022). Developments in the control of *D. suzukii* to limit the damage caused by this insect (Tait et al., 2021) could be considered in further studies for less insecticide-dependent control of the flies related to grape SR; these include biocontrol (using predators, parasitoids, or entomopathogens), mass trapping, attract, and kill, repellents, and oviposition repellents.

## Data Availability

The raw data supporting the conclusions of this article will be made available by the authors, without undue reservation.

## Footnotes

^1^www.wineseq.com

^2^www.biomemakers.com
