# Supplementary material for: The microbiome analysis of ripen grape berries supports the complex etiology of sour rot
Source: Front Microbiol. 2024 Nov 7;15:1450443. doi: 10.3389/fmicb.2024.1450443 (PMC11578972; doi:10.3389/fmicb.2024.1450443)
Supplement: Supplementary file 1 [file Table_1.DOCX]

Supplementary Material

The Microbiome Analysis of Ripen Grape Berries supports the Complex Etiology of Sour Rot

Chiara Brischetto^1^, Vittorio Rossi^1^, Giorgia Fedele^1*^

*** Correspondence:** Giorgia Fedele; [giorgiafedele@unicatt.it](mailto:giorgiafedele@unicatt.it)

# Supplementary Table and Figure

**Supplementary Table 1.** The numbers of reads for all sequence variants that were present in healthy and rotten bunches. Grape samples were collected from 39 vineyards in five Italian grape-growing regions (i.e., Veneto, Friuli Venezia Giulia, Emilia Romagna, Toscana, Lazio, and Puglia) in 2019, 2020, and 2021.

| **Fungi** | **Healthy** | **Affected** |
| --- | --- | --- |
| Acremonium | 11300 | 4772 |
| Acrodontium | 104 | 0 |
| Acrostalagmus | 107 | 0 |
| Agrocybe | 0 | 1 |
| Alternaria | 318072 | 70921 |
| Amanita | 4 | 0 |
| Amphinema | 35586 | 6271 |
| Amphobotrys | 0 | 6 |
| Angustimassarina | 55 | 415 |
| Apiosporina | 386 | 70 |
| Arachnomyces | 168 | 0 |
| Arthrographis | 0 | 1 |
| Articulospora | 10511 | 5249 |
| Ascochyta | 0 | 134 |
| Ascorhizoctonia | 0 | 58 |
| Aspergillus | 72087 | 128427 |
| Athelia | 65 | 0 |
| Aureobasidium | 211823 | 64421 |
| Auriculibuller | 0 | 54 |
| Bacidia | 286 | 0 |
| Baeospora | 10 | 0 |
| Bartalinia | 0 | 5 |
| Bimuria | 64 | 0 |
| Bipolaris | 0 | 6 |
| Bloxamia | 55 | 0 |
| Boeremia | 0 | 12 |
| Botryosphaeria | 14 | 7177 |
| Botryotinia | 143414 | 620486 |
| Botrytis | 132276 | 419393 |
| Bradymyces | 55 | 0 |
| Brettanomyces | 0 | 1 |
| Broomella | 0 | 53 |
| Buckleyzyma | 3588 | 616 |
| Bullera | 2912 | 556 |
| Bulleromyces | 1 | 2 |
| Cadophora | 12291 | 608 |
| Cadophora | 0 | 1963 |
| Calycina | 48 | 0 |
| Calyptella | 0 | 1 |
| Candida | 445926 | 1160230 |
| Cephaliophora | 299 | 0 |
| Ceratobasidium | 0 | 3 |
| Cercophora | 103 | 0 |
| Cercospora | 0 | 114 |
| Cerrena | 74 | 0 |
| Chaetomium | 0 | 4 |
| Chaetosphaeria | 0 | 1 |
| Chalara | 364 | 0 |
| Chalastospora | 1 | 0 |
| Chloridium | 126 | 1 |
| Chordomyces | 125 | 0 |
| Chrysosporium | 1194 | 309 |
| Ciboria | 0 | 103 |
| Cistella | 0 | 1 |
| Citeromyces | 0 | 123 |
| Cladophialophora | 29 | 2 |
| Cladosporium | 203640 | 57873 |
| Clathrus | 0 | 177 |
| Claviceps | 0 | 175 |
| Clavulina | 1 | 0 |
| Clitocella | 0 | 7 |
| Clohesyomyces | 44 | 2 |
| Clonostachys | 115 | 0 |
| Clonostachys | 0 | 1 |
| Colletotrichum | 2353 | 187 |
| Coniella | 0 | 1081 |
| Coniochaeta | 161 | 0 |
| Coniothyrium | 2329 | 3 |
| Conocybe | 44 | 0 |
| Coprinellus | 0 | 397 |
| Coprinopsis | 3964 | 0 |
| Cordyceps | 0 | 1 |
| Cortinarius | 1 | 14 |
| Crepidotus | 332 | 0 |
| Crocicreas | 101 | 0 |
| Cryptococcus | 16618 | 5667 |
| Cryptosporiopsis | 787 | 330 |
| Curvibasidium | 2117 | 1398 |
| Curvularia | 31903 | 367 |
| Cyphellophora | 0 | 84 |
| Cyphellophora | 0 | 66 |
| Cystobasidium | 0 | 66 |
| Cystodendron | 57 | 0 |
| Cystofilobasidium | 302 | 37 |
| Dactylonectria | 30 | 0 |
| Debaryomyces | 1296 | 61 |
| Devriesia | 71 | 322 |
| Didymella | 55181 | 14541 |
| Didymocyrtis | 322 | 0 |
| Didymosphaeria | 0 | 127 |
| Dioszegia | 5077 | 488 |
| Diplodia | 583 | 0 |
| Discohainesia | 0 | 100 |
| Dissoconium | 121541 | 8862 |
| Eleutherascus | 56 | 0 |
| Emmonsiellopsis | 113 | 0 |
| Endoconidioma | 0 | 30 |
| Epicoccum | 12793 | 5419 |
| Eremothecium | 1351 | 45 |
| Erysiphe | 9056 | 724 |
| Eupenicillium | 0 | 142 |
| Exophiala | 889 | 35 |
| Exserohilum | 0 | 251 |
| Extremus | 189 | 0 |
| Filobasidium | 58038 | 11214 |
| Fusarium | 2800 | 869 |
| Geminibasidium | 2299 | 251 |
| Genolevuria | 139 | 955 |
| Geotrichum | 0 | 139 |
| Glomerella | 27 | 0 |
| Gremmenia | 0 | 330 |
| Guehomyces | 21 | 0 |
| Gymnopus | 0 | 9 |
| Gyrothrix | 0 | 4 |
| Haematonectria | 189 | 0 |
| Hannaella | 450 | 1082 |
| Hanseniaspora | 474963 | 508307 |
| Hansfordia | 0 | 3 |
| Holtermannia | 75 | 13 |
| Holtermanniella | 0 | 88 |
| Hormonema | 16 | 0 |
| Humicola | 682 | 172 |
| Hymenoscyphus | 51 | 0 |
| Hyphodontia | 0 | 1 |
| Hypocrea | 1 | 1 |
| Idriella | 0 | 1 |
| Immersidiscosia | 0 | 190 |
| Inocybe | 59 | 75 |
| Issatchenkia | 2606 | 5429 |
| Itersonilia | 5388 | 0 |
| Kazachstania | 0 | 4 |
| Keissleriella | 3146 | 1905 |
| Kluyveromyces | 0 | 3 |
| Knufia | 262 | 0 |
| Kodamaea | 0 | 926 |
| Kwoniella | 570 | 0 |
| Lachancea | 206 | 206 |
| Lambertella | 0 | 19 |
| Lapidomyces | 57 | 0 |
| Latorua | 0 | 347 |
| Lecanicillium | 0 | 276 |
| Lecythophora | 2 | 1 |
| Leohumicola | 403 | 0 |
| Lepiota | 464 | 9 |
| Leptodiscella | 89 | 0 |
| Leptosphaeria | 1673 | 337 |
| Leptosphaerulina | 152 | 39 |
| Leptospora | 1 | 527 |
| Leuconeurospora | 56 | 0 |
| Lewia | 18458 | 5725 |
| Limonomyces | 0 | 4 |
| Lipomyces | 287 | 56 |
| Lophiotrema | 335 | 0 |
| Lycoperdon | 1 | 1 |
| Lysurus | 0 | 1 |
| Malassezia | 35431 | 3817 |
| Massarina | 55 | 0 |
| Metschnikowia | 72807 | 89616 |
| Meyerozyma | 10132 | 7435 |
| Microscypha | 370 | 0 |
| Microstroma | 0 | 2 |
| Monascus | 0 | 32 |
| Monilinia | 1 | 0 |
| Monocillium | 0 | 1 |
| Monographella | 166 | 58 |
| Mortierella | 27467 | 2270 |
| Mucor | 1 | 0 |
| Mycena | 263 | 0 |
| Mycenella | 125 | 0 |
| Mycocalicium | 0 | 521 |
| Mycoleptodiscus | 0 | 307 |
| Mycosphaerella | 654 | 318 |
| Myriodontium | 6277 | 0 |
| Myxotrichum | 61 | 0 |
| Naevala | 383 | 598 |
| Nakazawaea | 697 | 0 |
| Naohidea | 0 | 20 |
| Nemania | 72 | 0 |
| Neoascochyta | 0 | 209 |
| Neobulgaria | 1 | 1 |
| Neocucurbitaria | 5889 | 0 |
| Neodevriesia | 3363 | 411 |
| Neofusicoccum | 208 | 6171 |
| Neophaeococcomyces | 81 | 0 |
| Neophaeomoniella | 0 | 104 |
| Neosetophoma | 7315 | 1253 |
| Neosulcatispora | 72 | 0 |
| Nigrospora | 26 | 6 |
| Occultifur | 0 | 28 |
| Oidiodendron | 357 | 0 |
| Paecilomyces | 11 | 0 |
| Papiliotrema | 1377 | 339 |
| Paraconiothyrium | 976 | 48 |
| Paramycosphaerella | 0 | 145 |
| Paraphaeosphaeria | 45 | 7 |
| Paraphoma | 870 | 280 |
| Parastagonospora | 0 | 107 |
| Penicillago | 757 | 87 |
| Penicillium | 205351 | 148652 |
| Peniophora | 2078 | 98 |
| Periconia | 326 | 17 |
| Peroneutypa | 0 | 2 |
| Pestalotiopsis | 376 | 69 |
| Peziza | 1 | 0 |
| Phacidiella | 0 | 54 |
| Phaeococcomyces | 8228 | 1669 |
| Phaeomoniella | 0 | 3244 |
| Phaeopoacea | 0 | 162 |
| Phaeosphaeria | 1499 | 1428 |
| Phaeosphaeriopsis | 110 | 0 |
| Phallus | 11 | 25 |
| Phialocephala | 67 | 0 |
| Phialosimplex | 1 | 0 |
| Phlyctema | 0 | 1605 |
| Phoma | 394 | 25 |
| Phomopsis | 0 | 74 |
| Physcia | 0 | 189 |
| Pichia | 285 | 6995 |
| Pilidium | 54 | 0 |
| Pithoascus | 2208 | 0 |
| Plectosphaerella | 106 | 0 |
| Plenodomus | 282 | 280 |
| Pleospora | 14 | 2474 |
| Podosphaera | 76 | 122 |
| Podospora | 71 | 0 |
| Preussia | 12 | 0 |
| Psathyrella | 0 | 1 |
| Pseudallescheria | 0 | 1 |
| Pseudeurotium | 3530 | 383 |
| Pseudocamarosporium | 124 | 0 |
| Pseudogymnoascus | 579 | 123 |
| Pseudopithomyces | 2112 | 602 |
| Purpureocillium | 82 | 0 |
| Pyrenochaetopsis | 258 | 222 |
| Pyrenophora | 1642 | 314 |
| Radulidium | 880 | 4804 |
| Ramichloridium | 8994 | 2128 |
| Ramularia | 103944 | 4937 |
| Rhizopus | 0 | 159 |
| Rhodosporidiobolus | 2204 | 374 |
| Rhodosporidium | 27239 | 936 |
| Rhodotorula | 3159 | 1659 |
| Roussoella | 0 | 1 |
| Russula | 182 | 0 |
| Saccharomyces | 709 | 1807 |
| Saccharomycodes | 0 | 135 |
| Saccharomycopsis | 2379 | 21712 |
| Sagenomella | 98 | 0 |
| Saitozyma | 62 | 191 |
| Sampaiozyma | 58 | 0 |
| Sarocladium | 14976 | 3688 |
| Schizothecium | 0 | 60 |
| Schizothyrium | 4283 | 242 |
| Schwanniomyces | 2643 | 11289 |
| Scolecobasidium | 0 | 1 |
| Scutellinia | 112 | 0 |
| Sebacina | 6 | 18 |
| Seimatosporium | 0 | 134 |
| Septoria | 76 | 227 |
| Serendipita | 0 | 1 |
| Setophoma | 69 | 1 |
| Simocybe | 0 | 1 |
| Solicoccozyma | 6193 | 332 |
| Spiromastix | 0 | 1 |
| Sporobolomyces | 23018 | 11500 |
| Stachybotrys | 226 | 0 |
| Stagonospora | 1264 | 92 |
| Stagonosporopsis | 102 | 109 |
| Starmera | 0 | 22931 |
| Starmerella | 352112 | 1025384 |
| Stemphylium | 6071 | 4093 |
| Stephanonectria | 0 | 123 |
| Suhomyces | 0 | 57 |
| Symmetrospora | 22454 | 3397 |
| Talaromyces | 1076 | 39248 |
| Tausonia | 107 | 0 |
| Tetracladium | 804 | 92 |
| Titaea | 60 | 0 |
| Tomentella | 1661 | 370 |
| Torula | 0 | 15 |
| Torulaspora | 0 | 3175 |
| Trechispora | 0 | 2 |
| Tremateia | 171 | 236 |
| Tricellula | 7600 | 0 |
| Trichoderma | 103 | 53 |
| Tricholoma | 363 | 0 |
| Trichopeziza | 1 | 0 |
| Trichosporon | 0 | 129 |
| Trichothecium | 0 | 149 |
| Trigonopsis | 82 | 0 |
| Truncatella | 0 | 1 |
| Tuber | 9758 | 1685 |
| Umbelopsis | 286 | 0 |
| Uncinula | 26451 | 1200 |
| Uncobasidium | 0 | 30 |
| Uwebraunia | 971 | 8 |
| Verticillium | 280 | 281 |
| Vishniacozyma | 49160 | 7496 |
| Volutella | 0 | 1 |
| Vuilleminia | 0 | 187 |
| Wallemia | 135 | 162 |
| Wardomyces | 16 | 0 |
| Wickerhamomyces | 0 | 18 |
| Xenasmatella | 0 | 9 |
| Xenomeris | 0 | 11 |
| Yamadazyma | 0 | 50 |
| Zasmidium | 73 | 0 |
| Zetiasplozna | 62 | 74 |
| Zygoascus | 998 | 84363 |
| Zygosaccharomyces | 1546 | 36161 |
| Zygosporium | 215 | 0 |
| Zygotorulaspora | 0 | 225 |
| Zymoseptoria | 5491 | 0 |
| **Bacteria** |  |  |
| Abditibacterium | 3 | 9 |
| Acetobacter | 2286 | 29308 |
| Achromobacter | 2062 | 3882 |
| Acidibacter | 1478 | 515 |
| Acidicaldus | 376 | 109 |
| Acidiphilium | 231 | 0 |
| Acidomonas | 0 | 30 |
| Acidothermus | 3727 | 1874 |
| Acidovorax | 15074 | 3105 |
| Acinetobacter | 16261 | 2037 |
| Actinomyces | 0 | 9 |
| Actinomycetospora | 16 | 0 |
| Adhaeribacter | 581 | 2 |
| Advenella | 52 | 104 |
| Aerococcus | 3 | 0 |
| Aeromicrobium | 0 | 9 |
| Aeromonas | 0 | 2 |
| Afipia | 30834 | 384 |
| Agrobacterium | 1062 | 124 |
| Agrococcus | 3 | 0 |
| Agromyces | 1 | 86 |
| Akkermansia | 464 | 0 |
| Alcanivorax | 1898 | 1063 |
| Algoriphagus | 6 | 12 |
| Alicyclobacillus | 0 | 2 |
| Alishewanella | 0 | 2 |
| Alistipes | 613 | 55 |
| Alkalihalobacillus | 12630 | 2058 |
| Alkaliphilus | 0 | 6 |
| Alkanindiges | 53 | 0 |
| Allochromatium | 8 | 0 |
| Allorhizobium-Neorhizobium-Pararhizobium-Rhizobium | 6268 | 696 |
| Altererythrobacter | 212 | 5 |
| Alysiosphaera | 0 | 22 |
| Amaricoccus | 0 | 8 |
| Aminobacter | 0 | 804 |
| Ammoniphilus | 0 | 29 |
| Amnibacterium | 95 | 3 |
| Anaerobacillus | 20873 | 1265 |
| Anaerococcus | 2954 | 0 |
| Anaeromyxobacter | 0 | 12 |
| Anaerostipes | 0 | 300 |
| Aquamicrobium | 2 | 3 |
| Aquicella | 0 | 109 |
| Aquipuribacter | 2 | 0 |
| Aquisphaera | 582 | 59 |
| Arcobacter | 235 | 60 |
| Arcticibacter | 52 | 7 |
| Arenimonas | 63 | 0 |
| Arsenophonus | 0 | 794 |
| Arthrobacter | 1459 | 650 |
| Asaia | 43 | 831 |
| Aureimonas | 5415 | 1796 |
| Azoarcus | 340 | 0 |
| Azospira | 132 | 129 |
| Bacillus | 155825 | 33217 |
| Bacteriovorax | 0 | 13 |
| Bacteroides | 34250 | 15359 |
| Balneimonas | 0 | 14 |
| Bauldia | 52 | 5 |
| Bdellovibrio | 1553 | 291 |
| Belnapia | 100 | 0 |
| Bifidobacterium | 381 | 79 |
| Blastocatella | 2 | 357 |
| Blastococcus | 111 | 9 |
| Blastomonas | 104 | 0 |
| Blastopirellula | 283 | 11 |
| Blattabacterium | 2610 | 0 |
| Blautia | 0 | 15 |
| Bosea | 0 | 2 |
| Brachybacterium | 0 | 88 |
| Bradyrhizobium | 5 | 93 |
| Brevibacterium | 0 | 130 |
| Brevifollis | 54 | 0 |
| Brevinema | 0 | 6 |
| Brevundimonas | 1992 | 388 |
| Brochothrix | 2725 | 1305 |
| Brownia | 0 | 32 |
| Bryobacter | 3132 | 1513 |
| Burkholderia | 633 | 52 |
| Burkholderia-Caballeronia-Paraburkholderia | 1971 | 1569 |
| Butyrivibrio | 0 | 5 |
| Byssovorax | 29 | 8 |
| Bythopirellula | 122 | 3 |
| Caenimonas | 4 | 58 |
| Caldibacillus | 2 | 0 |
| Caldilinea | 1229 | 4 |
| Calothrix | 355 | 0 |
| Campylobacter | 0 | 15 |
| Cardinium | 0 | 13 |
| Carnimonas | 0 | 754 |
| Carnobacterium | 158 | 0 |
| Catellatospora | 1 | 0 |
| Catellicoccus | 0 | 21 |
| Caulobacter | 1 | 2 |
| Cellulomonas | 26 | 1 |
| Cellvibrio | 0 | 25 |
| Chishuiella | 0 | 3087 |
| Chitinophaga | 1 | 71 |
| Chryseobacterium | 9485 | 1785 |
| Chryseolinea | 0 | 11 |
| Chthoniobacter | 2074 | 760 |
| Chthonomonas | 216 | 50 |
| Citrobacter | 717 | 254 |
| Clavibacter | 5 | 21 |
| Cloacibacterium | 126 | 0 |
| Clostridium | 2166 | 1496 |
| Cohnella | 0 | 134 |
| Comamonas | 67 | 10 |
| Commensalibacter | 0 | 135 |
| Competibacter | 282 | 0 |
| Compostimonas | 45 | 0 |
| Conexibacter | 1917 | 619 |
| Coprococcus | 0 | 1 |
| Coprothermobacter | 0 | 32 |
| Corynebacterium | 6885 | 148 |
| Curtobacterium | 15873 | 1887 |
| Curvibacter | 82 | 0 |
| Cutibacterium | 1141 | 2303 |
| Dechloromonas | 0 | 140 |
| Deinococcus | 0 | 11 |
| Desulfovibrio | 61 | 9 |
| Devosia | 763 | 131 |
| Dokdonella | 228 | 0 |
| Dolosigranulum | 1389 | 1 |
| Dongia | 0 | 57 |
| Duganella | 770 | 293 |
| Dyadobacter | 1483 | 204 |
| Dyella | 1561 | 680 |
| Dysgonomonas | 0 | 10 |
| Edaphobaculum | 1636 | 533 |
| Empedobacter | 0 | 1505 |
| Enhydrobacter | 27104 | 1 |
| Enterobacter | 23503 | 2797 |
| Enterococcus | 1682 | 607 |
| Entomoplasma | 0 | 18 |
| Entotheonella | 0 | 23 |
| Erwinia | 176 | 64 |
| Erysipelothrix | 3632 | 996 |
| Escherichia | 2613 | 1868 |
| Escherichia-Shigella | 1649 | 463 |
| Exiguobacterium | 1 | 10 |
| Facklamia | 5 | 0 |
| Faecalibacterium | 0 | 19 |
| Faecalibaculum | 353 | 78 |
| Falsirhodobacter | 0 | 24 |
| Ferruginibacter | 900 | 320 |
| Fibrobacter | 0 | 1 |
| Finegoldia | 0 | 250 |
| Flavirhabdus | 84 | 104 |
| Flavisolibacter | 2251 | 1427 |
| Flavobacterium | 5191 | 1079 |
| Fluviicola | 68 | 19 |
| Fontibacter | 1 | 3 |
| Fonticella | 17 | 0 |
| Frateuria | 15697 | 10124 |
| Friedmanniella | 0 | 2 |
| Frigoribacterium | 31 | 99 |
| Fructobacillus | 122 | 20700 |
| Gaiella | 2935 | 1708 |
| Gemmata | 1601 | 427 |
| Gemmatimonas | 2199 | 749 |
| Geobacillus | 0 | 18 |
| Georgenia | 7 | 0 |
| Gilliamella | 0 | 8423 |
| Gluconacetobacter | 53997 | 127774 |
| Gluconobacter | 184833 | 312613 |
| Gordonia | 51 | 0 |
| Granulicatella | 3 | 0 |
| Granulicella | 220 | 0 |
| Halalkalibacillus | 1 | 1 |
| Haliangium | 1330 | 474 |
| Haliea | 2 | 0 |
| Haloferula | 0 | 2 |
| Halomonas | 33647 | 6419 |
| Hepatincola | 0 | 48 |
| Herminiimonas | 0 | 23 |
| Hymenobacter | 44368 | 15876 |
| Hyphomicrobium | 650 | 165 |
| Iamia | 733 | 130 |
| Ideonella | 16 | 0 |
| Ignatzschineria | 0 | 3 |
| Ilumatobacter | 0 | 5 |
| Isosphaera | 0 | 1 |
| Izhakiella | 323 | 220 |
| Janibacter | 71 | 0 |
| Janthinobacterium | 2113 | 173 |
| Jatrophihabitans | 1150 | 276 |
| Kaistobacter | 160 | 26 |
| Kineococcus | 251 | 105 |
| Klebsiella | 12066 | 3239 |
| Klenkia | 1645 | 65 |
| Kocuria | 506 | 2 |
| Komagataeibacter | 65436 | 456313 |
| Koribacter | 1015 | 118 |
| Kosakonia | 68 | 0 |
| Ktedonobacter | 3 | 2 |
| Kurthia | 992 | 247 |
| Lactobacillus | 12788 | 8724 |
| Lactococcus | 22226 | 11133 |
| Lacunisphaera | 76 | 0 |
| Lautropia | 9 | 0 |
| Legionella | 1135 | 0 |
| Leifsonia | 1478 | 0 |
| Leptolyngbya | 1 | 0 |
| Leuconostoc | 20475 | 8029 |
| Levilactobacillus | 0 | 37 |
| Limnobacter | 3 | 0 |
| Listeria | 0 | 20 |
| Litorilinea | 0 | 1 |
| Longilinea | 0 | 2 |
| Luedemannella | 0 | 1 |
| Luteibacter | 0 | 5 |
| Luteolibacter | 1234 | 237 |
| Lutispora | 93 | 19 |
| Lysinibacillus | 73 | 312 |
| Lysobacter | 5381 | 483 |
| Marmoricola | 80 | 0 |
| Massilia | 57521 | 20934 |
| Melittangium | 0 | 9 |
| Mesorhizobium | 130 | 21 |
| Methylobacterium | 60237 | 26216 |
| Methylobacterium-Methylorubrum | 28885 | 4085 |
| Methylocystis | 0 | 9 |
| Methylorubrum | 9774 | 3900 |
| Methylotenera | 372 | 33 |
| Microbacterium | 910 | 467 |
| Microbispora | 0 | 1 |
| Micrococcus | 13989 | 28 |
| Microlunatus | 0 | 17 |
| Micromonospora | 291 | 0 |
| Microterricola | 8255 | 6161 |
| Microthrix | 1 | 7 |
| Mitsuaria | 58 | 0 |
| Moranella | 0 | 598 |
| Moraxella | 1889 | 648 |
| Morganella | 91 | 3514 |
| Mucilaginibacter | 6015 | 2030 |
| Mycetocola | 5 | 5 |
| Mycobacterium | 116 | 103 |
| Mycolicibacterium | 105 | 0 |
| Nakamurella | 115 | 0 |
| Nannocystis | 0 | 6 |
| Neisseria | 5199 | 0 |
| Neoasaia | 341 | 0 |
| Neobacillus | 1559 | 756 |
| Neokomagataea | 0 | 1037 |
| Niastella | 63 | 0 |
| Nitrosococcus | 304 | 39 |
| Nitrosomonas | 590 | 54 |
| Nitrososphaera | 763 | 12 |
| Nitrospira | 52 | 72 |
| Nitrotoga | 929 | 190 |
| Nocardioides | 1581 | 448 |
| Nocardiopsis | 19 | 0 |
| Nordella | 1 | 0 |
| Noviherbaspirillum | 18 | 45 |
| Novosphingobium | 1347 | 527 |
| Occallatibacter | 53 | 0 |
| Oceanobacillus | 0 | 6 |
| Ochrobactrum | 1 | 314 |
| Oenococcus | 1343 | 1 |
| Opitutus | 425 | 3 |
| Orbus | 22935 | 195800 |
| Ornatilinea | 0 | 3 |
| Ornithinimicrobium | 0 | 1 |
| Oryzihumus | 56 | 1 |
| Owenweeksia | 0 | 1 |
| Paenibacillus | 4821 | 5311 |
| Pajaroellobacter | 124 | 0 |
| Pantoea | 107031 | 24726 |
| Parabacteroides | 176 | 7 |
| Paraburkholderia | 494 | 114 |
| Paracoccus | 274 | 393 |
| Parafrigoribacterium | 8 | 0 |
| Parasegetibacter | 0 | 18 |
| Patulibacter | 0 | 13 |
| Pectobacterium | 110 | 612 |
| Pediococcus | 2360 | 1322 |
| Pedobacter | 13889 | 12794 |
| Pedomicrobium | 260 | 57 |
| Pedosphaera | 1 | 4 |
| Pelosinus | 1 | 7 |
| Peptoclostridium | 68 | 0 |
| Peptoniphilus | 0 | 25 |
| Persicitalea | 1 | 0 |
| Phaselicystis | 50 | 6 |
| Phenylobacterium | 5004 | 1 |
| Phormidium | 117 | 0 |
| Photobacterium | 2071 | 1210 |
| Phycisphaera | 1 | 2 |
| Phyllobacterium | 663 | 64 |
| Pirellula | 1178 | 302 |
| Planctomyces | 2 | 8 |
| Plantibacter | 59 | 21 |
| Polaromonas | 26 | 0 |
| Pontibacter | 0 | 2 |
| Porphyrobacter | 54 | 0 |
| Prevotella_9 | 666 | 0 |
| Pricia | 1 | 0 |
| Prochlorococcus | 493 | 120 |
| Propionibacterium | 748 | 138 |
| Prosthecobacter | 371 | 0 |
| Proteus | 0 | 147 |
| Providencia | 0 | 372 |
| Pseudarcicella | 69 | 0 |
| Pseudarthrobacter | 6144 | 3393 |
| Pseudoalteromonas | 848 | 178 |
| Pseudoclavibacter | 0 | 35 |
| Pseudolabrys | 2358 | 1006 |
| Pseudomonas | 34556 | 15862 |
| Pseudonocardia | 51 | 72 |
| Pseudoxanthomonas | 444 | 4 |
| Puia | 514 | 203 |
| Pusillimonas | 1 | 0 |
| Quadrisphaera | 177 | 0 |
| Rahnella | 103 | 1402 |
| Ralstonia | 2802 | 133 |
| Ramlibacter | 1121 | 354 |
| Rathayibacter | 2020 | 397 |
| Reyranella | 618 | 118 |
| Rhizobacter | 293 | 0 |
| Rhizobium | 4587 | 1185 |
| Rhizomicrobium | 1 | 0 |
| Rhodanobacter | 889 | 156 |
| Rhodobium | 1 | 0 |
| Rhodococcus | 1033 | 17 |
| Rhodopirellula | 0 | 5 |
| Rhodoplanes | 1753 | 694 |
| Rhodopseudomonas | 0 | 51 |
| Rickettsia | 687 | 219 |
| Rickettsiella | 1 | 6 |
| Robinsoniella | 5 | 0 |
| Romboutsia | 0 | 2081 |
| Roseiflexus | 119 | 8 |
| Roseimicrobium | 0 | 1 |
| Roseomonas | 669 | 156 |
| Rothia | 51 | 0 |
| Rubellimicrobium | 0 | 2 |
| Rubrobacter | 172 | 88 |
| Ruminococcus | 55 | 7 |
| Saccharimonas | 0 | 7 |
| Salana | 0 | 3 |
| Salmonella | 0 | 7 |
| Schumannella | 0 | 94 |
| Sedimentibacter | 0 | 7 |
| Sediminibacterium | 1044 | 139 |
| Segetibacter | 52 | 1 |
| Serratia | 0 | 55 |
| Shimazuella | 0 | 7 |
| Siccationidurans | 111 | 0 |
| Singulisphaera | 618 | 32 |
| Skermanella | 58 | 64 |
| Sodalis | 8690 | 3736 |
| Solibacter | 4057 | 1784 |
| Solirubrobacter | 276 | 50 |
| Soonwooa | 145 | 0 |
| Sorangium | 0 | 13 |
| Sphingoaurantiacus | 227 | 3 |
| Sphingobacterium | 184 | 6531 |
| Sphingobium | 195 | 23 |
| Sphingomonas | 190128 | 60684 |
| Spiroplasma | 0 | 67 |
| Spirosoma | 2451 | 713 |
| Sporosarcina | 1 | 0 |
| Staphylococcus | 18787 | 7850 |
| Stenotrophobacter | 938 | 232 |
| Stenotrophomonas | 1945 | 419 |
| Steroidobacter | 0 | 3 |
| Streptococcus | 3956 | 1087 |
| Streptomyces | 1402 | 563 |
| Sufflavibacter | 236 | 0 |
| Sulcia | 26819 | 11753 |
| Sulfurihydrogenibium | 52 | 0 |
| Taibaiella | 0 | 14512 |
| Tanticharoenia | 6754 | 13365 |
| Tatumella | 122837 | 42779 |
| Telmatobacter | 1 | 3 |
| Terracidiphilus | 63 | 0 |
| Terriglobus | 315 | 17 |
| Terrimonas | 920 | 137 |
| Thermoactinomyces | 0 | 5 |
| Thermobacillus | 2 | 4 |
| Thiothrix | 0 | 1 |
| Trabulsiella | 1156 | 0 |
| Tremblaya | 0 | 29 |
| Trichococcus | 0 | 1 |
| Truepera | 218 | 549 |
| Tumebacillus | 0 | 18 |
| Tundrisphaera | 1362 | 0 |
| Udaeobacter | 4149 | 1807 |
| Variovorax | 13314 | 4340 |
| Verticiella | 668 | 252 |
| Wautersiella | 0 | 1440 |
| Weissella | 210 | 165 |
| Williamsia | 0 | 3 |
| Wohlfahrtiimonas | 0 | 2 |
| Wolbachia | 1965 | 10467 |
| Woodsholea | 0 | 1 |
| Xanthomonas | 357 | 2 |
| Xiphinematobacter | 5 | 454 |
| Xylophilus | 0 | 5 |
| Yersinia | 7510 | 4750 |
| Yonghaparkia | 407 | 389 |
| Zymobacter | 0 | 1777 |

# Supplementary Figure


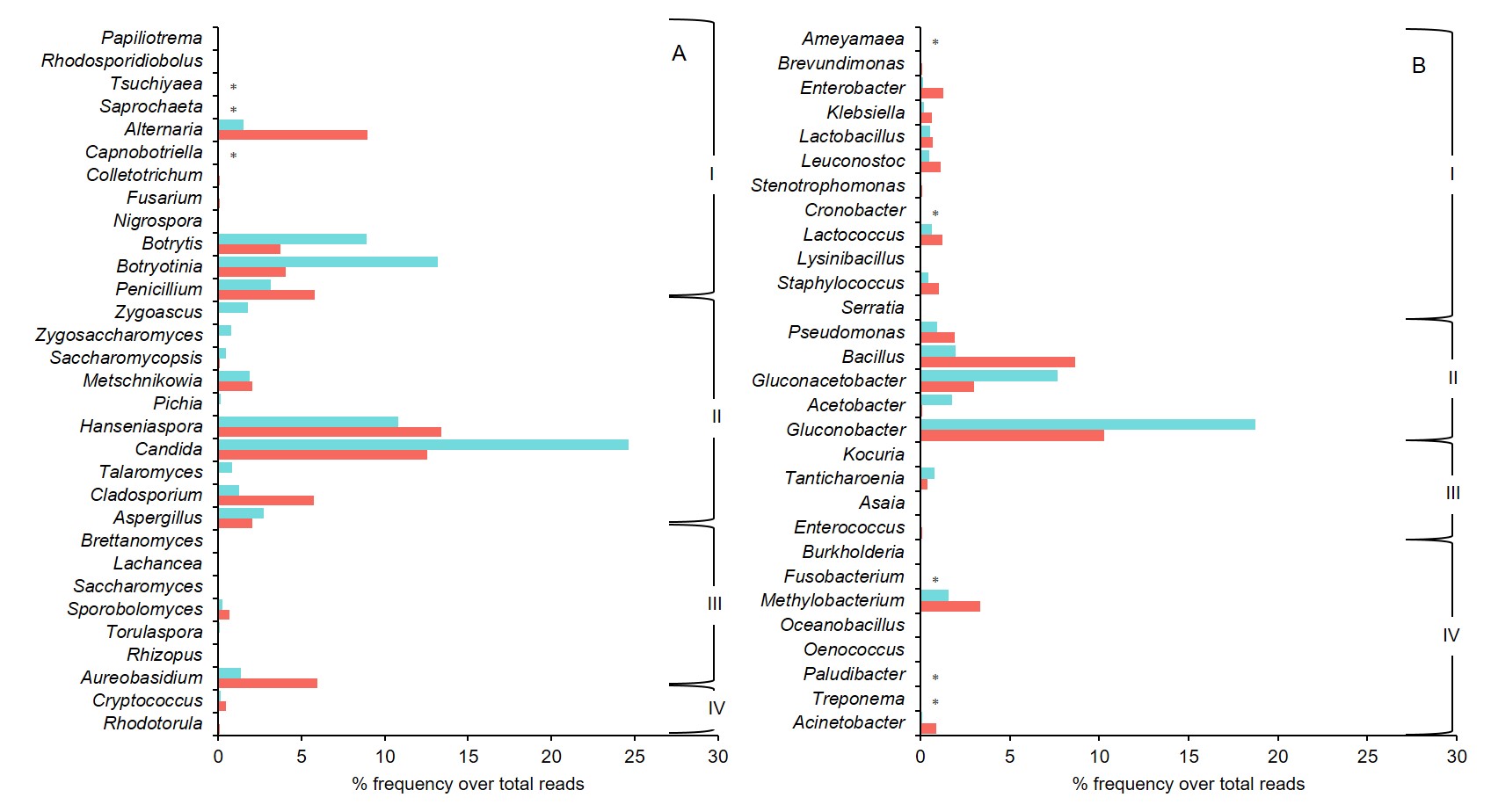


**Supplementary Figure 1.** The abundance of some fungal (A) and bacterial (B) genera in grapevine bunches showing sour rot symptoms (light blue) or not (i.e., healthy, red), which have been found in the previous literature on SR (Brischetto et al., 2024) in rotten bunches only (I), more in rotten than in healthy bunches (II), in both healthy and rotten bunches with a similar frequency (III), and healthy bunches only (IV). The symbol * means that the genus was not found in our samples.

Reference

Brischetto, C., Rossi, V., and Fedele, G. (2024). Knowledge gaps on grape sour rot inferred from a systematic literature review. *Frontiers in Plant Science*, 15, 1415379.
